# The Shock-Induced Deformation and Spallation Failure of Bicrystal Copper with a Nanoscale Helium Bubble via Molecular Dynamics Simulations

**DOI:** 10.3390/nano13162308

**Published:** 2023-08-11

**Authors:** Qi Zhu, Jianli Shao, Pei Wang

**Affiliations:** 1 State Key Laboratory of Explosion Science and Technology, Beijing Institute of Technology, Beijing 100081, China; 2 Explosion Protection and Emergency Disposal Technology Engineering Research Center of the Ministry of Education, Beijing 100039, China; 3 Institute of Applied Physics and Computational Mathematics, Beijing 100094, China; 4 Center for Applied Physics and Technology, Peking University, Beijing 100871, China

**Keywords:** helium bubble, grain boundary, shock compression, spallation, molecular dynamics

## Abstract

Both the nanoscale helium (He) bubble and grain boundaries (GBs) play important roles in the dynamic mechanical behavior of irradiated nanocrystalline materials. Using molecular dynamics simulations, we study the shock-induced deformation and spallation failure of bicrystal copper with a nanoscale He bubble. Two extreme loading directions (perpendicular or parallel to the GB plane) and various impact velocities (0.5–2.5 km/s) are considered. Our results reveal that the He bubble shows hindrance to the propagation of shock waves at lower impact velocities but will accelerate shock wave propagation at higher impact velocities due to the local compression wave generated by the collapse of the He bubble. The parallel loading direction is found to have a greater effect on He bubble deformation during shock compression. The He bubble will slightly reduce the spall strength of the material at lower impact velocities but has a limited effect on the spallation process, which is dominated by the evolution of the GB. At lower impact velocities, the mechanism of spall damage is dominated by the cleavage fracture along the GB plane for the perpendicular loading condition but dominated by the He bubble expansion and void growth for the parallel loading condition. At higher impact velocities, micro-spallation occurs for both loading conditions, and the effects of GBs and He bubbles can be ignored.

## 1. Introduction

Nanocrystalline materials have attracted extensive attention due to their excellent radiation resistance. There exist various defects, such as dislocations, voids, grain boundaries (GBs), and, especially, helium (He) bubbles, in irradiated nanocrystalline materials [1,2,3,4]. Both the He bubbles and GBs significantly affect the mechanical properties of these materials. For example, the existence of a He bubble may lead to local hardening [5,6], embrittlement [7,8], and swelling [9,10] of the materials. Also, the GB was found to play an important role in the plasticity and fracture behavior of the materials. Additionally, the GB was verified to be a good trap for He atoms, leading to the massive formation of He bubbles inside it [11,12,13]. Therefore, it is of scientific and practical significance to study the coupling effect of the He bubble and the GB on the mechanical behavior of the materials.

To date, many efforts have been devoted to exploring the influence of He bubbles on the dynamic mechanical behavior of these materials. Experimentally, Glam et al. [14,15] carried out planar impact experiments on Al-^10^B samples. They found that the spall strength of the sample with He bubbles is lower than the bubble-free one at 600 °C but is similar for both cases at room temperature. Using MD simulations, the plastic deformation of a He bubble and the surrounding matrix in single-crystal Al under dynamic loadings was investigated. It was found that the dislocation nucleation on the leading side of the He bubble is easier than that of the void under shock compression, and stacking fault tetrahedral was formed during the collapse of He bubble during uniform compression [16,17]. Also, it was investigated that He bubbles can greatly influence the shock response of Cu, including the Hugoniot elastic limit, deformation mechanisms, bubble collapse, and surface jetting [18,19]. Shock-induced deformation changes the microstructure of the material, thereby affecting its behaviors during tensile loading, such as the spallation behaviors. It was verified that He bubbles provide preferential starting sites for damage evolution during spallation and can significantly reduce the spall strength. Recently, Zhou et al. [20] found that the expansion and coalescence of He bubbles dominate the spallation behavior of a ductile metal with He bubbles.

The GBs have been acknowledged to play a crucial role in the mechanical properties of nanocrystalline materials. For example, GB-mediated plasticity is assumed to dominate the ductility and fracture behavior of nanocrystalline materials [21,22,23,24,25,26,27]. Both intragranular and intergranular fracture behaviors were observed during tension, which can be highly dependent on grain sizes, GB characteristics, and loading conditions. Fensin et al. [28] investigated the effect of the loading direction with respect to the GB on the material’s failure behavior under shock loading and found that the GBs perpendicular to the loading direction are more susceptible to failure than those parallel to the loading direction.

Undoubtedly, both the He bubbles and GBs play important roles in the dynamic mechanical behavior of irradiated nanocrystalline materials. However, as reviewed above, most simulations in previous studies are limited to a system containing a single defect (only a He bubble or a GB). As for a He bubble in a GB, the related research is relatively limited. Recently, we studied the dynamic response of He bubbles in bicrystal Cu under uniaxial compression and tension and revealed the effect of He bubble positions (in grain interiors and GBs) for different loading orientations [29]. However, the coupling effect of a He bubble and a GB on the mechanical behavior under shock loading has not been addressed exclusively.

Compared with the conventional nanocrystalline material, the bicrystal is simpler in structure, which makes the coupling effect between the He bubble and the GB beneficial to explore. In this work, we perform MD simulations in bicrystal copper with a nanoscale He bubble to investigate the dynamic evolution of the He bubble and the GB during shock compression and spallation. The effects of shock orientations relative to the GB (perpendicular or parallel) and shock intensities are discussed through detailed analysis of microstructures and mechanical quantities. All these findings are considered instructive for understanding the dynamic mechanical behavior of He bubbles in irradiated nanocrystalline metals. This paper is organized as follows. Section 2 introduces the computational details, then the simulation results are presented and discussed in Section 3, followed by the conclusions in Section 4.

## 2. Computational Details

The MD simulations are carried out using the Large-scale Atomic/Molecular Massively Parallel Simulator (LAMMPS) [30]. The embedded-atom-method (EAM) potential developed by Mishin et al. [31] is used to describe the atomic interactions between Cu. Both Cu-He and He-He interatomic interactions are described by the Lennard-Jones potentials, which can be described as follows:

(1) $$\emptyset \left(r\right)=4\varepsilon \left[{\left(\frac{\sigma }{r}\right)}^{a}-{\left(\frac{\sigma }{r}\right)}^{b}\right]$$

where *a* = 9, *b* = 6, *ε* = 0.000745 eV, and *σ* = 3.546 Å for the Cu-He interaction [32], and *a* = 12, *b* = 6, *ε* = 0.000876 eV, and *σ* = 2.280 Å for the He-He interaction [33]. The selected potentials have been demonstrated to accurately reflect the structural and physical characteristics of Cu containing a He bubble [34,35].

**Figure 1:**
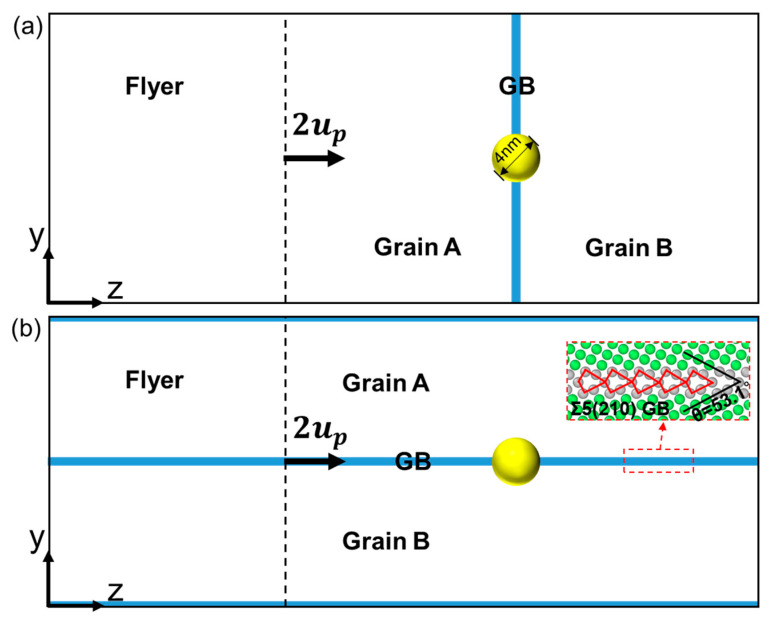
Simulation setup. (**a**) Initial configuration corresponding to the perpendicular loading condition; and (**b**) initial configuration corresponding to the parallel loading condition. The yellow ball refers to He bubble.

Figure 1 shows the details of the simulation setup. The bicrystal is created by rotating grain A and grain B along the [1 0 0] crystallographic orientation using the software ATOMSK (Version 0.11) [36]. The initial configuration is approximately 31.6 nm × 31.7 nm × 77.9 nm in dimension and contains about 6.5 × 10^6^ atoms. To investigate the influence of loading orientations relative to the GB plane, we consider two extreme loading directions: perpendicular and parallel to the GB plane, as shown in Figure 1a,b, respectively. The GB between grain A ([1 0 0], [0 1 $\bar{2}$], [0 2 1]) and grain B ([1 0 0], [0 2 $\bar{1}$], [0 1 2]) is a symmetrical-tilt high-energy GB ($\gamma_{GB}=953\;mJ/m^{2}$) with a misorientation angle of 53.1°, namely, Σ5{210}, which consists of continuous structural units, as highlighted in red in Figure 1b. It has been investigated in a previous study that high-energy GBs provide more effective sinks for irradiation-induced defects [37]. A He bubble (4 nm in diameter) is placed in the central region of the GB plane by removing Cu atoms in a spherical region and then adding He atoms into this region. And the He/vacancy ratio of the He bubble is set to 1.0. The diameter and He/vacancy ratio of the He bubble are determined based on previous experimental observations [38,39] and simulations [40,41], where the He bubbles in the GBs were larger than those in the grain interiors. All the simulation parameters selected in our models ensure that the drawn conclusions are robust.

Before loading, the bicrystal model is first relaxed by an energy minimization procedure using the standard conjugate gradient method and then optimization at 300 K and 0 bar pressure using the isothermal isobaric (NPT) ensemble under 3D periodic boundary conditions for 100 ps to obtain a stable, stress-free configuration. The integration time step is set as 1 fs. The shock-loading simulations are carried out under the microcanonical (NVE) ensemble, and the free boundary condition is applied in the shock direction (*z*-axis direction). As shown in Figure 1a,b, the bicrystal is divided into two regions: flyer and target. The velocity of atoms in the flyer is set to 2$u_{p}$ ($u_{p}$ is the particle velocity after shock) along the *z*-axis direction, initially. Then, two planar shock waves are generated and propagate towards the flyer and target, respectively. After reaching the free surface, two rarefaction waves reflected from the left and right surfaces propagate towards each other, resulting in spallation failure. It should be noted that the target region is twice as long as the flyer region in our models. Such a setting ensures that spallation can occur in the center of the sample, which enables a better study of the effect of the He bubble and GB.

The adaptive common neighbor analysis (a-CNA) [42] and the center-symmetry parameter (CSP) in the open-access software OVITO (Version 3.0.0) [43] are utilized to analyze the microstructure evolution in the system. The stress is calculated based on the virial theorem [44], and the temperature is calculated by subtracting the center-of-mass velocity from the average kinetic energy of each bin.

## 3. Results and Discussion

### 3.1. Deformation Mechanism under Shock Compression

The deformation mechanism under shock compression has a significant influence on the subsequent spallation behavior. In this subsection, the evolution properties during shock compression, including the shock-wave evolution, microstructural evolution, and He bubble evolution, are discussed in detail.

#### 3.1.1. Shock-Wave Evolution

**Figure 2:**
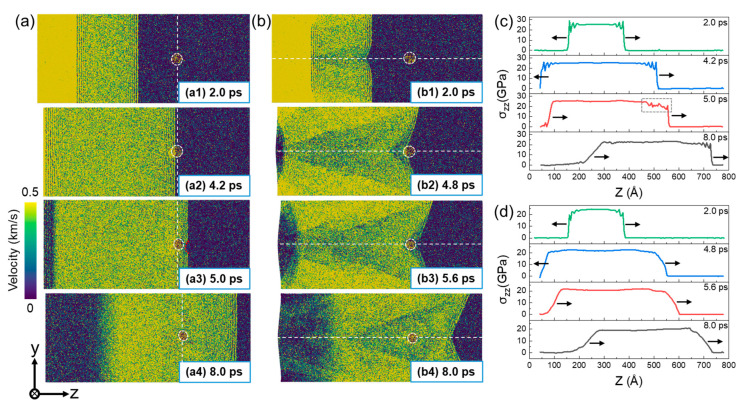
The distribution of particle velocity (**a**,**b**) and corresponding stress evolution (**c**,**d**) of a Σ5(210) GB system at $u_{p}$ = 0.5 km/s. (**a**,**c**): Perpendicular loading condition; and (**b**,**d**): parallel loading condition. The atoms are color-coded by the velocity along the shock direction. The white dotted lines indicate the positions of He bubble and GB. The black arrow indicates the direction of wave propagation.

The presence of preexisting defects (He bubble and GB) directly affects the shock-wave evolution during shock compression. Figure 2 shows the two-dimensional (2D) distribution of particle velocity and corresponding stress evolution for a Σ5(210) GB system at $u_{p}$ = 0.5 km/s. For the perpendicular loading condition, the generated plane shock wave propagates uniformly to the Cu matrix. At t = 4.2 ps, the shock wave interacts with the rear surface of the He bubble, followed by the interaction with the GB. It can be seen that the shock wave just passing through the He bubble region propagates slightly slower than that in other regions, as the red dotted line in Figure 2(a3) shows, indicating the hindering effect of the He bubble on shock-wave propagation. Also, the existence of both the GB and He bubble causes disturbances in the stress evolution, where the stress decreases slightly after passing through that region, as indicated by the gray dotted box in Figure 2c. For the parallel loading condition, due to the effect of the GB, the shock wave propagates nonuniformly to the Cu matrix, that is, the wave near the GB is slightly slower than that in other regions. Similarly, the impediment of the He bubble to the shock-wave propagation is observed. It should be noted that this impediment is rather weak due to the small He bubble in this work and can be ignored as the shock wave propagates further, as Figure 2(a4,b4) show.

**Figure 3:**
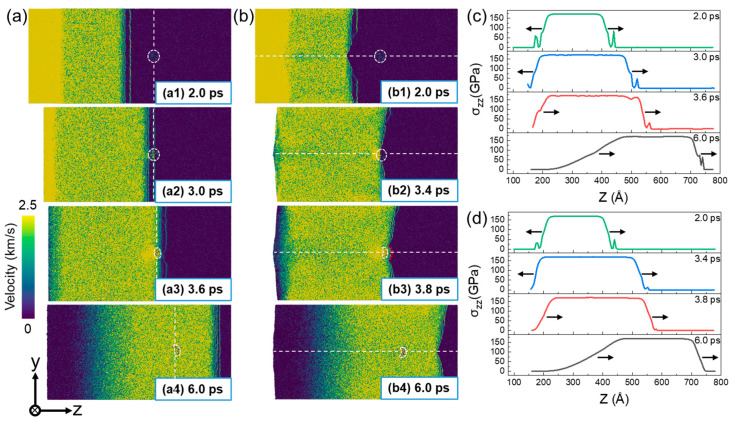
The distribution of particle velocity (**a**,**b**) and corresponding stress evolution (**c**,**d**) of a Σ5(210) GB system at $u_{p}$ = 2.5 km/s. (**a**,**c**): Perpendicular loading condition; and (**b**,**d**): parallel loading condition. The atoms are color-coded by the velocity along the shock direction. The white dotted lines indicate the positions of He bubble and GB. The black arrow indicates the direction of wave propagation.

Figure 3 presents the two-dimensional (2D) distribution of atomic velocity and corresponding stress evolution of a Σ5(210) GB system at $u_{p}$ = 2.5 km/s. At this impact velocity, an obvious double-wave structure is observed in the stress profile, since the elastic wave propagates faster than the plastic wave. Normally, the He bubble exhibits a significant hindrance to the propagation of an elastic precursor wave. However, as the plastic wave interacts with the He bubble, the shock wave propagates faster in the He bubble than in the matrix, and an obvious shock-wave acceleration is observed at the front of the He bubble (see Figure 3(a3,b3)). This may be due to the internal jetting mechanism of the He bubble when subjected to high pressure. At high impact velocities, the collapse of the He bubble occurs and generates a local compression wave that propagates towards the matrix, leading to acceleration of the propagation of waves around it.

#### 3.1.2. Microstructural Evolution during Compression

**Figure 4:**
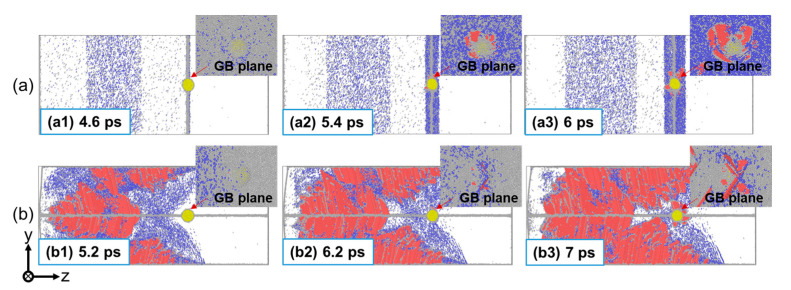
The microstructure evolution of a Σ5(210) GB system at $u_{p}$ = 0.5 km/s. (**a**) Perpendicular loading condition; and (**b**) parallel loading condition. He atoms are colored in yellow and other atoms are colored based on a-CNA (adaptive common neighbor analysis), where the red, blue, and gray atoms correspond to HCP, BCC, and disorder atoms, respectively.

The microstructure evolution of a Σ5(210) GB system at $u_{p}$ = 0.5 km/s is presented in Figure 4. For the perpendicular loading condition, the shock wave interacts with the GB plane and He bubble at t = 4.6 ps. As a result, the structure of the GB plane has been destroyed, which becomes highly disordered, accompanied by the generation of a number of BCC atoms. As shown in Figure 4(a2), a large number of BCC atoms are observed near the GB plane. Simultaneously, dislocations begin to be emitted from the junction of the He bubble and GB plane. At t = 6 ps, after a period of shock-wave action, the dislocations are emitted from other regions of the GB plane. Then, with the development of plasticity, the generated dislocations will propagate towards the grain interiors on both sides. For the parallel loading condition, a large number of defects (stacking faults and dislocations) are formed near the GB plane as the shock wave passes through it, as shown in Figure 4(b1). Similarly, the dislocations will be emitted from the junction of the He bubble and GB plane and then propagate towards the grain interiors on both sides to interact with the dislocations from the GB plane. The deformation behavior of the material is consistent with the distribution of the shock wave, as exhibited in Figure 2 and Figure 3, where the particle velocity is symmetrically distributed with respect to the GB.

**Figure 5:**
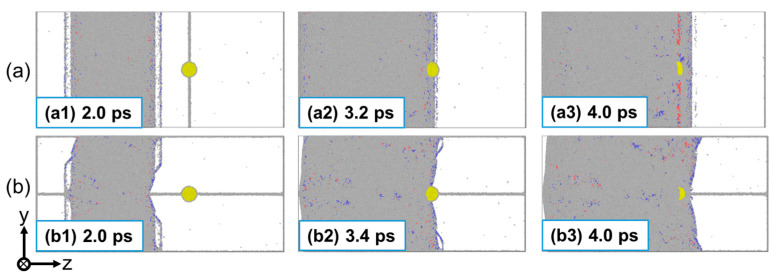
The microstructure evolution of a Σ5(210) GB system at $u_{p}$ = 2.5 km/s. (**a**) Perpendicular loading condition; and (**b**) parallel loading condition. He atoms are colored in yellow and other atoms are colored based on a-CNA (adaptive common neighbor analysis), where the red, blue, and gray atoms correspond to HCP, BCC, and disorder atoms, respectively.

Figure 5 presents the microstructure evolution of a Σ5(210) GB system at $u_{p}$ = 2.5 km/s. For both perpendicular and parallel loading conditions, a great number of disordered atoms are generated under shock compression, rather than the regular stacking faults and dislocations under a lower impact velocity. At this impact velocity, the crystal structure of the material is completely destroyed and amorphization occurs. Consequently, the effects of GBs and He bubbles can be neglected under such a strong shock.

#### 3.1.3. He Bubble Evolution during Compression

**Figure 6:**
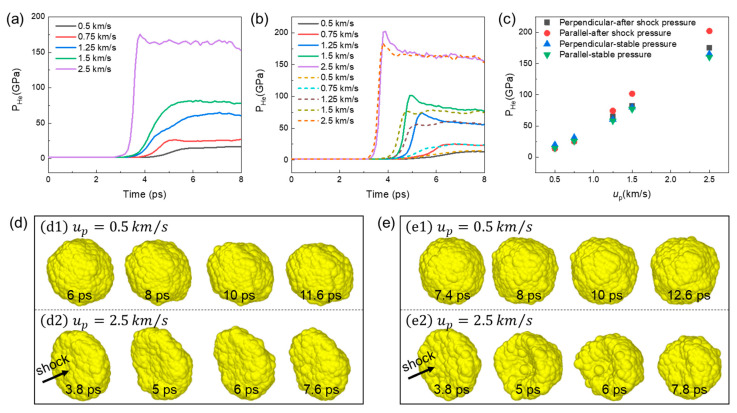
The pressure (**a**–**c**) and shape (**d**,**e**) evolution of a He bubble under different impact velocities. (**a**) Bubble pressure evolution for perpendicular loading condition; (**b**) bubble pressure evolution for parallel loading condition; (**c**) bubble pressure after shock and after reaching stability; (**d**) bubble shape evolution for perpendicular loading condition; and (**e**) bubble shape evolution for parallel loading condition. The dashed curves in (**b**) indicate the pressure evolution of the He bubble in the grain interior.

The He bubble evolution under different loading conditions is discussed in this subsection. Figure 6 illustrates the pressure and shape evolution of a He bubble in a Σ5(210) GB system under different impact velocities. As shown in Figure 6a,b, the evolution of the He bubble pressure during shock compression can also be divided into three stages: keeping constant before interacting with the shock wave (stage I), increasing rapidly during interaction (stage II), and reaching stability after shock (stage III). At stage II, the shock wave propagates to interact with the He bubble and induces the rapid increase in He bubble pressure. It can be seen that the growth rate of He bubble pressure increases with the impact velocity, regardless of the loading direction. Specifically, at lower impact velocities, the pressure of the He bubble keeps increasing for a period of time after the shock wave. However, at higher impact velocities, the pressure of the He bubble increases rapidly and reaches a peak value in a very short time after the shock wave due to the strong shock pressure. It is observed that the growth rate of He bubble pressure is greater in the parallel loading condition than that in the perpendicular loading condition for higher impact velocities ($u_{p}$ ≥ 1.25 km/s). At stage III, the pressure of the He bubble keeps a slight increase at lower impact velocities, such as in the cases when $u_{p}$ = 0.5 km/s and $u_{p}$ = 0.75 km/s. This can be due to the plastic deformation evolution around the He bubble. As the impact velocity further increases, the peak pressure after shock is unstable and will decrease to a stable value in subsequent evolution. It should be pointed out that this phenomenon is more significant under the parallel loading condition, as shown in Figure 6b. Figure 6c presents the pressure of the He bubble after shock (stage II) and after reaching stability (stage III). It can be seen that after-shock pressure and stable pressure increase with the impact velocity. The stable pressure for two loading conditions is similar at the same impact velocity. However, there are differences in the bubble pressure just after the shock wave. It is observed the after-shock pressure in the parallel loading condition is obviously higher than that in the perpendicular loading condition for higher impact velocities. Also, the differences between the after-shock pressure and stable pressure increase with the impact velocity, which is also more obvious for the parallel loading condition. For comparison, the pressure evolution of the He bubble in the grain interior under the parallel shock compression is also plotted in Figure 6b. It is observed that the peak pressure of He bubbles in the grain interior is lower than that in the GB, indicating that it is the GB that leads to more significant pressure fluctuations. Figure 6d,e illustrate the typical shape evolution of He bubbles during shock compression when $u_{p}$ = 0.5 km/s and $u_{p}$ = 2.5 km/s. Apparently, the deformation of He bubbles becomes more severe as the impact velocity increases. At $u_{p}$ = 0.5 km/s, the He bubble remains spherical and only slight deformation near the bubble surface is observed for both the perpendicular and parallel loading conditions. As $u_{p}$ increases to 2.5 km/s, the shape of the He bubble changes violently during shock compression. It can be seen from Figure 6(d2,e2) that the He bubble is partially collapsed along the compression direction as the shock wave passes through it. The degree of He bubble collapse is more pronounced under the parallel loading condition, where the obvious depression of the He bubble along the loading direction can be observed (Figure 6(e2)). Then, after a period of evolution, the He bubble will return to an irregular reduced sphere under the action of the Cu-He interface.

**Figure 7:**
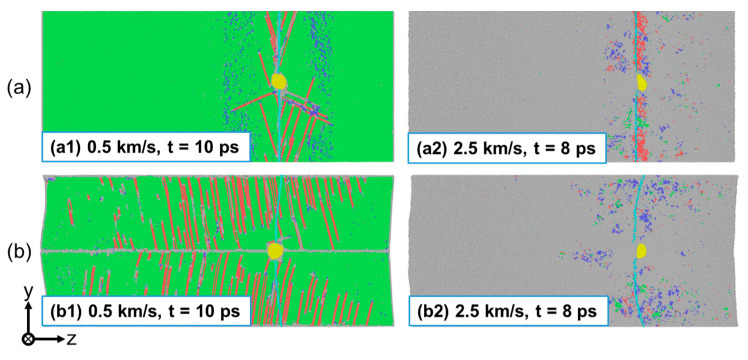
The migration of a He bubble during shock compression. (**a**) Perpendicular loading condition; and (**b**) parallel loading condition. He atoms are colored in yellow and other atoms are colored based on a-CNA (adaptive common neighbor analysis), where the green, red, blue, and gray atoms correspond to FCC, HCP, BCC, and disorder atoms, respectively. The light-blue atomic lines indicate the Cu atoms passing through the center of the He bubble at the initial moment.

Under strong shock, the migration of the He bubble may occur. Figure 7 shows the position of the He bubble relative to the Cu matrix after shock compression. The light-blue atomic lines in Figure 7 indicate the Cu atoms passing through the center of the He bubble at the initial moment. At $u_{p}$ = 0.5 km/s, there is no significant difference in the position of He bubbles relative to the matrix before and after impact, as shown in Figure 7(a1,b1). However, at $u_{p}$ = 2.5 km/s, obvious migration of the He bubble is observed for both loading conditions, as seen in Figure 7(a2,b2). This is not surprising, since the He bubble will obtain significant acceleration during the collapse under strong shock, which will result in the following inertial motion. The shock-induced migration of the He bubble has also been observed in single-crystal Al [45]. Here, the migration distance in the two loading conditions is similar, indicating that the GB has little effect on He bubble migration at such a high loading velocity.

### 3.2. Spallation Behavior

Spallation occurs when the rarefaction waves reflected from both free surfaces interact with each other and generate strong tensile stress. The dynamic failure process of spallation is usually accompanied by the nucleation, growth, and aggregation of microdamages (usually voids or cracks) in the material. The initial defects (He bubble and GB) and their evolution during shock compression may greatly influence this failure process. In this subsection, we investigate the spallation behaviors of bicrystal copper with a He bubble by analyzing the strength and damage evolution process. Three typical impact velocities ($u_{p}$ = 0.5 km/s, 1.5 km/s, and 2.5 km/s) are selected for detailed analysis to reveal the different degrees and mechanisms of He bubbles and GBs in influencing the spallation behaviors at different velocities.

#### 3.2.1. Spall Strength

**Figure 8:**
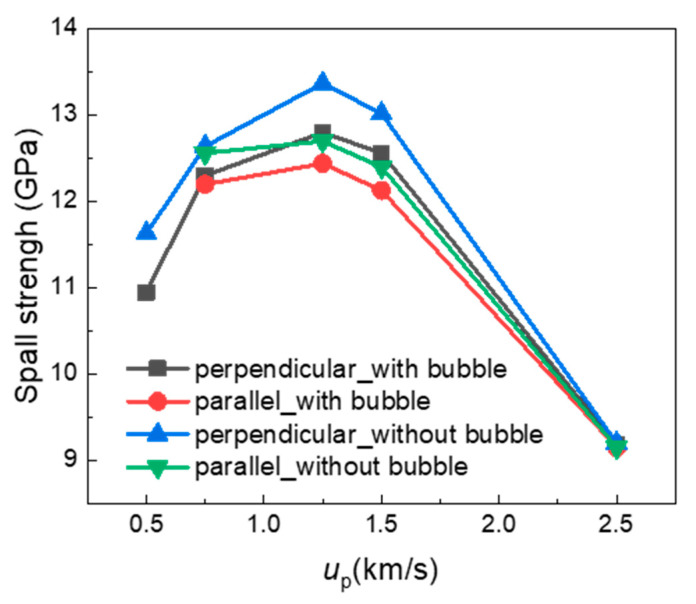
Spall strengths at various impact velocities based on the maximum tensile stress during the tensile process.

The spall strength is a key property for estimating the resistance to spall damage of the material. Figure 8 exhibits the spall strength at various impact velocities based on the maximum tensile stress in the sample during the tensile process. For comparison, the spall strength of a sample without a He bubble is also plotted in Figure 8. It is observed that the spall strength shows an increase–decrease trend with the impact velocity for all four samples. Specifically, the spall strength increases with the increasing impact velocity when $u_{p}$ ≤ 1.25 km/s but decreases with the increasing impact velocity as $u_{p}$ goes higher. This agrees well with previous studies on the spallation of FCC metals and can be attributed to competition between the strain rate effect and temperature softening effect. For $u_{p}$ ≤ 1.5 km/s, the spallation strength of samples with a He bubble is lower than the bubble-free ones for both perpendicular and parallel loading conditions. This suggests that the existence of a He bubble will reduce the spall strength of the bicrystal system at lower impact velocities. As $u_{p}$ increases to 2.5 km/s, the spall strength of the four samples is almost the same (about 9.1 GPa), indicating the effect of the He bubble on spall strength can be ignored at strong shock intensities. Note that the spall strength in Figure 8 refers to the maximum tensile stress value in the whole sample. Thus, we can see that the spall strength under perpendicular loading is higher than that under the parallel loading condition for lower impact velocities, due to a more concentrated damaged zone and less defect generation in non-GB regions for the perpendicular loading condition. The mechanism will be analyzed in detail below.

#### 3.2.2. Damage Evolution Process

**Figure 9:**
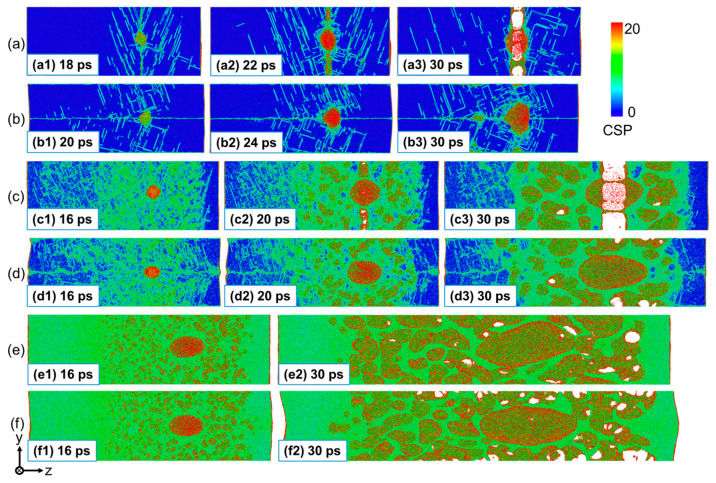
Microstructures during spallation of the Σ5(210) GB system with a He bubble at different impact directions and velocities. (**a**) Perpendicular shock at $u_{p}$ = 0.5 km/s; (**b**) parallel shock at $u_{p}$ = 0.5 km/s; (**c**) perpendicular shock at $u_{p}$ = 1.5 km/s; (**d**) parallel shock at $u_{p}$ = 1.5 km/s; (**e**) perpendicular shock at $u_{p}$ = 2.5 km/s; and (**f**) parallel shock at $u_{p}$ = 2.5 km/s. The atoms are colored based on CSP (center-symmetry parameter).

Figure 9 details the spallation process at three typical impact velocities ($u_{p}$ = 0.5 km/s, 1.5 km/s, and 2.5 km/s). Different morphologies in the damaged zone are observed for different loading directions and impact velocities. At $u_{p}$ = 0.5 km/s, there exist a number of defects (dislocations and stacking faults) around the GB plane at the initial stage of tension, which evolve from the defects formed during the early compression process for both the perpendicular and parallel loading conditions, as shown in Figure 9(a1,b1). Then, the He bubble expands, accompanied by the dislocations further propagating into the surrounding matrix under tensile stress. The difference is that a number of voids are formed in the GB plane for the perpendicular loading condition (Figure 9(a2)), and these voids will grow and coalesce rapidly, causing a complete fracture of the material at the GB plane (Figure 9(a3)). However, for the parallel loading condition, only the expansion of the He bubble but not void nucleation is observed until t = 24 ps (Figure 9(b2)). At t = 30 ps, the He bubble expands more, and a small number of voids are observed at the GBs, but no spallation occurs in the material (Figure 9(b3)). At $u_{p}$ = 1.5 km/s, the shock-induced deformation is much more severe, causing a much wider defect area, which provides wider nucleation sites for voids. It can be seen that the nucleation of voids occurs not only in the GB plane but also in the grain interiors (Figure 9(c2,d2)). At this impact velocity, classical spallation failure along the GB plane occurs for the perpendicular loading condition, while micro-spallation occurs for the parallel loading condition. At $u_{p}$ = 2.5 km/s, homogeneous void nucleation becomes dominant in the spallation process. Micro-spallation occurs for both loading conditions and there is little difference in the morphology of the damaged zone, as can be seen in Figure 9(e2,f2). The result suggests that the effect of the GB on the spallation behaviors can be ignored at strong shock intensities. In conclusion, for the perpendicular loading condition, the mechanism of spall damage is dominated by the cleavage fracture along the GB plane at weaker shock intensities but by the void nucleation–growth–coalescence in the wider damage zone at stronger shock intensities. For the parallel loading condition, the classical spallation failure hardly occurs due to energy dissipation during shock compression and He bubble expansion.

**Figure 10:**
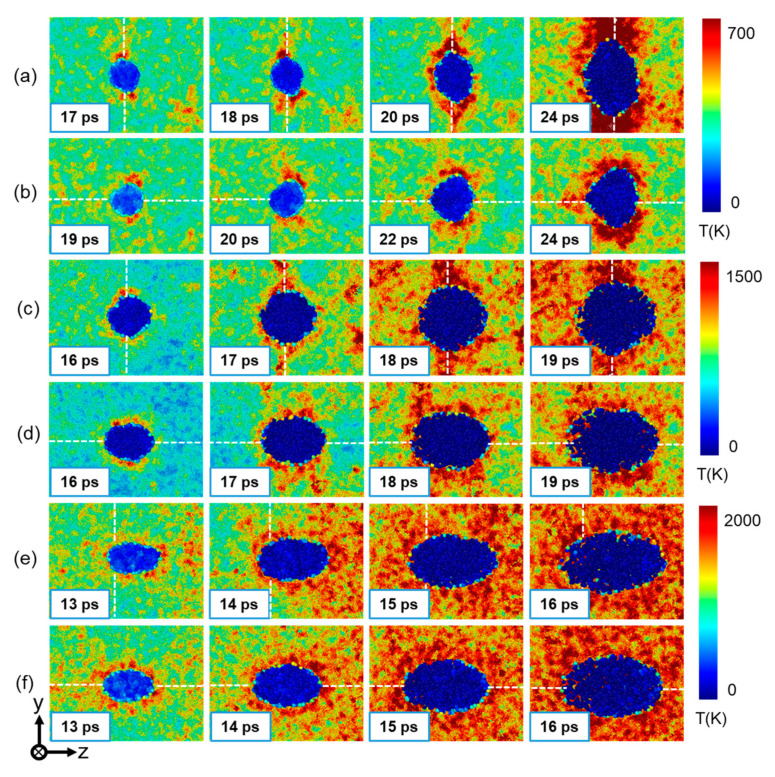
Local temperature evolution around the He bubble in the Σ5(210) GB system at different impact directions and velocities. (**a**) Perpendicular shock at $u_{p}$ = 0.5 km/s; (**b**) parallel shock at $u_{p}$ = 0.5 km/s; (**c**) perpendicular shock at $u_{p}$ = 1.5 km/s; (**d**) parallel shock at $u_{p}$ = 1.5 km/s; (**e**) perpendicular shock at $u_{p}$ = 2.5 km/s; and (**f**) parallel shock at $u_{p}$ = 2.5 km/s. The atoms are colored according to the temperature. The white dotted lines indicate the positions of GB.

The local temperature evolution of the matrix around the He bubble at different impact directions and velocities is shown in Figure 10. The temperature of each atom is calculated by averaging its neighbors inside a spherical 5 angstroms. Obvious temperature rise can be observed in the nearby matrix during the expansion process of the He bubble. It is observed that the initial temperature-rising distribution is uneven under lower impact velocities, where the direction of He bubble expansion shows a higher local temperature rise, as shown in Figure 10a–d. This uneven-local-temperature-rise characteristic can be due to the anisotropic development of plastic deformation at lower impact velocities, which dominates the growth of the He bubble. At $u_{p}$ = 2.5 km/s, uniform temperature rise is observed in the matrix around the He bubble, since the Cu atoms around the He bubble are highly disordered and local melting occurs under such strong shock intensity, as shown in Figure 10e,f. In addition, as the impact velocity increases, the shape evolution of the He bubble transforms from transverse deformation to longitudinal deformation along the tensile direction under both loading conditions. Specifically, at $u_{p}$ = 0.5 km/s, the expansion of the He bubble along the y direction is much greater than that along the z direction, which is opposite to that when $u_{p}$ = 2.5 km/s. In addition, the loading condition will influence the shape evolution of the He bubble due to the effect of the GB, which is more pronounced under a low impact velocity. At $u_{p}$ = 0.5 km/s, the transverse deformation of the He bubble is accelerated by the GB for the perpendicular loading condition (see Figure 10a). However, for the parallel loading condition, the transverse deformation of the He bubble is suppressed as compared to the perpendicular loading, and expansion along the GB is observed (see Figure 10b). Also, longitudinal deformation of the He bubble along the tensile direction is more pronounced under parallel loading (see Figure 10c,d). As $u_{p}$ increases to 2.5 km/s, the shape evolution of the He bubble is almost the same under the two loading conditions, which deforms along the tensile direction, indicating that the effect of the GB can be ignored at this impact velocity.

## 4. Conclusions

In this work, MD simulations are performed on bicrystal Cu with a nanoscale He bubble to study the effects of shock orientations relative to the GB (perpendicular or parallel) and shock intensities on the dynamic compression deformation and spallation failure behaviors of the material. Our MD simulations reveal that the He bubble shows hindrance to the propagation of shock waves at lower impact velocities but will accelerate shock-wave propagation at higher impact velocities due to the local compression wave generated by the collapse of the He bubble. Compared to perpendicular loading, the parallel loading direction shows greater effect on He bubble deformation under shock compression, in both the pressure and shape evolution. The spallation of the bicrystal system is dominated by the GB rather than the He bubble. At lower impact velocities, the He bubble is found to slightly reduce the spall strength of the material but has limited effect on the spallation process. The mechanism of spall damage is dominated by the cleavage fracture along the GB plane for the perpendicular loading condition but dominated by He bubble expansion and void growth for the parallel loading condition. At higher impact velocities, void nucleation–growth–coalescence in the large damage zone occurs and leads to the micro-spallation of the materials for both loading conditions. Additionally, the loading conditions will affect the shape evolution of the He bubble during spallation, i.e., the perpendicular loading condition accelerates the transverse deformation of the He bubble, whereas the parallel loading condition promotes longitudinal deformation of the He bubble. It should be pointed out that the conclusions here are obtained from the bicrystal sys-tem with a specific GB (Σ5{210}) and a specific size He bubble (4 nm). As for the more complex irradiated polycrystalline systems, which contain various types of GBs and He bubbles of various sizes, the degree of the influence may be slightly different. However, the related physical mechanism is similar and this work provides a reference for understanding the dynamic mechanical behavior of irradiated nanocrystalline metals.

## Data Availability

The data presented in this study are available on request from the corresponding author.
